# A soil productivity system reveals most Brazilian agricultural lands are below their maximum potential

**DOI:** 10.1038/s41598-023-39981-y

**Published:** 2023-08-29

**Authors:** Lucas T. Greschuk, José A. M. Demattê, Nélida E. Q. Silvero, Nícolas Augusto Rosin

**Affiliations:** Department of Soil Science, University of São Paulo (ESALQ/USP), Av. Pádua Dias, 11, Piracicaba, SP 13418-900 Brazil

**Keywords:** Environmental social sciences, Plant sciences

## Abstract

Food production is extremely dependent on the soil. Brazil plays an important role in the global food production chain. Although only 30% of the total Brazilian agricultural areas are used for crop and livestock, the full soil production potential needs to be evaluated due to the environmental and legal impossibility to expand agriculture to new areas. A novel approach to assess the productive potential of soils, called “SoilPP” and based on soil analysis (0–100 cm) - which express its pedological information - and machine learning is presented. Historical yields of sugarcane and soybeans were analyzed, allowing to identify where it is still possible to improve crop yields. The soybean yields were below the estimated SoilPP in 46% of Brazilian counties and could be improved by proper management practices. For sugarcane, 38% of areas can be improved. This technique allowed us to understand and map the food yield situation over large areas, which can support farmers, consultants, industries, policymakers, and world food security planning.

## Introduction

The continuous demand for food and fuel increases the pressure on agricultural areas around the world^1^. Strategies to face these demands have led to improvements in food production by optimizing natural resources that are still available for agriculture^2,3^. Brazil is one of the most important producers and exporters of agricultural products in the world^4^, with 30% of its territory (263 million hectares) used for agricultural activities^5^. Agribusiness contributed approximately 27% to the Brazilian GDP in 2020, with soybean and sugarcane being the main commodities produced^4^. According to FAOSTAT^4^, Brazil was the world's largest producer of soybeans (134.9 million tons) and sugarcane (715.7 million tons) in 2021. Therefore, Brazil's importance and competitiveness in agricultural production on the world is remarkable. Although there is still room for agricultural expansion, some authors suggested that this is not considered a viable option to meet future food demands^6^. New sustainable strategies should be developed to improve yields where crops are already grown, which will allow optimizing the use of natural resources^7,8^, especially the soil, which is under constant anthropogenic pressure^9^.

Soils have an inherent capacity to meet crop demands in terms of availability of nutrients and water, influencing the photosynthetic process of the plant and consequently the production of biomass, which can be defined as their productive potential (SoilPP). There are some examples of measuring the productive potential of agricultural lands. Vogel et al.^10^ assessed the potential of German soils at the farm level to produce wheat by using water availability and soil texture as indicators. Huang et al.^11^ evaluated the effect of texture and soil organic carbon (SOC) concentration (indicators) on yield responses of seven major crops grown in the United States of America from 1958 to 2019. They found that crop yields were higher in soils with fine texture and higher SOC concentration, due to increased available water, soil structure, and nutrient retention^11^. In Brazil, there is a method named Production Environment System “PES”^12–16^ based on soil texture^10,11^, soil fertility^17^, water retention^11,18^, soil depth^18^, and soil classification^19^. The combination of all these factors gives an empirical classification system varying from A (high potential) to G (low potential), which has been in use in the last 30 years to understand the soil’s potential to produce sugarcane^12,16^. In conclusion, the soil pedological maps express the soil solution dynamics, drived by horizons A (surface) and B (undersurface) and thus, impact on soil productivity.

The information on soil’s potential to produce biomass can help us understand where agricultural yields can still be improved. This information is also helpful for policymaking. An example is the ‘Iowa Corn Adaptability Rating for Your Farm’^20^, in which corn yield is estimated in bags/acre based on soil information, terrain properties, and soil classification. This strategy can assist in agricultural resource management, variable rate application which reduces input use and environmental impact, crop planning, and soil security^21–23^ helping to balance food yield between countries. Mellor^24^ indicated that to understand policy needs and potentials, it is necessary to understand the underlying nature of current global food imbalances. Therefore, knowing the potential of soils could bring to light several opportunities such as the understanding and optimization of agricultural systems. However, there is a need for techniques that focus on the systematic measurement of continental extensions on a fine scale, with analysis of subsurface soils, as indicated by Bishopp and Lynch^18^. Although SoilPP can be estimated through the evaluation of its chemical, physical and biological properties^25^, there is a lack of knowledge about the soil’s potential to produce biomass and whether it can be used as an indicator to identify if crop yield can be increased.

This is a challenging task in large territorial extensions with remarkable differences in biomes, climate, and soil, typical characteristics of the Brazilian territory^19,25,26^. An alternative to fill this gap is the Digital Soil Mapping (DSM) framework, which is considered a useful tool for working with spatial predictions, making it possible to predict soil properties at global scales^27^. DSM is defined as the *“creation and population of spatial soil information systems by numerical models inferring the spatial and temporal variations of soil types and soil properties from soil observation and knowledge and from related environmental variables”*^28^. DSM approaches are specifically used to obtain spatial maps of physical, chemical and biological soil properties or soil classes, which can be used in conjunction with scoring methods^21,29,30^ to spatially assess and quantify soil’s intrinsic potential^10^.

In this study, a strategy to map the productive potential of Brazilian agricultural soils at a fine-scale resolution (30 m) was developed, corresponding to approximately 260 M ha. Information on soil properties and multivariate statistics was used to build a scoring system named SoilPP, which varied from 0 to 100 (that express the pedological information of the site), higher values representing high potential of soils to produce biomass. The SoilPP was evaluated against historical sugarcane and soybean yield at the county level, considering the economic importance of these two crops.

## Results

### The soil productive potential - SoilPP

**Figure 1:**
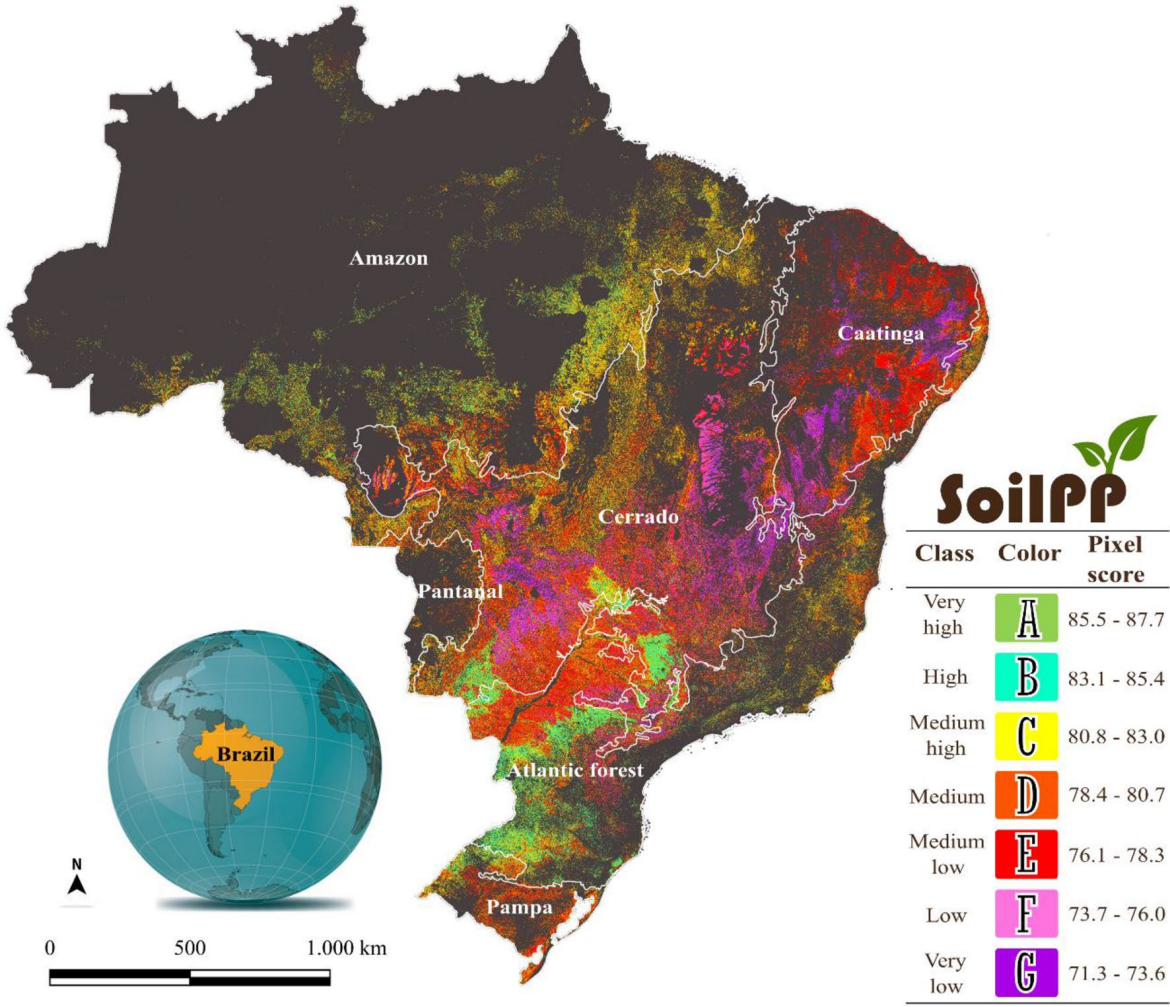
The Soil Productive Potential (SoilPP) map for Brazilian agricultural areas is classified into seven groups from A to G, representing very high and very low productive potentials. SoilPP*score*: Soil Productive Potential score (pixel value). The figure was created by the software QGIS Development Team (2021). Version 3.22. http://qgis.osgeo.org and Inkscape Project (2021). Version 1.1. https://inkscape.org.

The productive potential of Brazilian agricultural areas (SoilPP map) up to 1 m depth divided into seven classes (A through G) is depicted in Fig. 1 and represents about 203 million hectares (M ha). The agricultural areas (pixels) with the highest SoilPP values (A, B, and C classes) are the best in terms of productive potential. Classes E, F, and G are those that have the lowest values of the SoilPP score. Soils belonging to class 'A' (SoilPP: very high) (Fig. 1) feature greater depth, good drainage, fine texture, and sufficient nutrients for plants. The relief can be variable but without the presence of rocky outcrops. Conversely, the main characteristics of soils that belong to class 'G' (SoilPP: very low) are the low capacity to provide nutrients to plants, coarse texture (sandy), low water retention, and consequently low availability for the plant, varied depth and, in some cases, high presence of stones. Categories B and C' have a greater similarity with class 'A', while categories 'E and F' are closer to class 'G'.

The Brazilian agricultural soils presented a very high SoilPP (class A) represented by the green color (Fig. 1), corresponding to approximately 11.1 M ha (5% of the total agricultural area). The high SoilPP (class B) had an area of 15 M ha (7% of the total) while the medium–high SoilPP (class C) corresponded to 45 M ha (22% of the total). These are the agricultural areas that have the greatest productive potential, corresponding to about 34% of the total agricultural area. The medium SoilPP areas occupied approximately 50 M ha (24.5% of the total area). Areas with medium/low, low, and very low SoilPP occupied 44.1, 20.8, and 18.5 M ha representing 21.5, 10, and 9% of the total agricultural area, respectively. The uses of agricultural crops and pasture were considered in this representation (Fig. 1).

**Figure 2:**
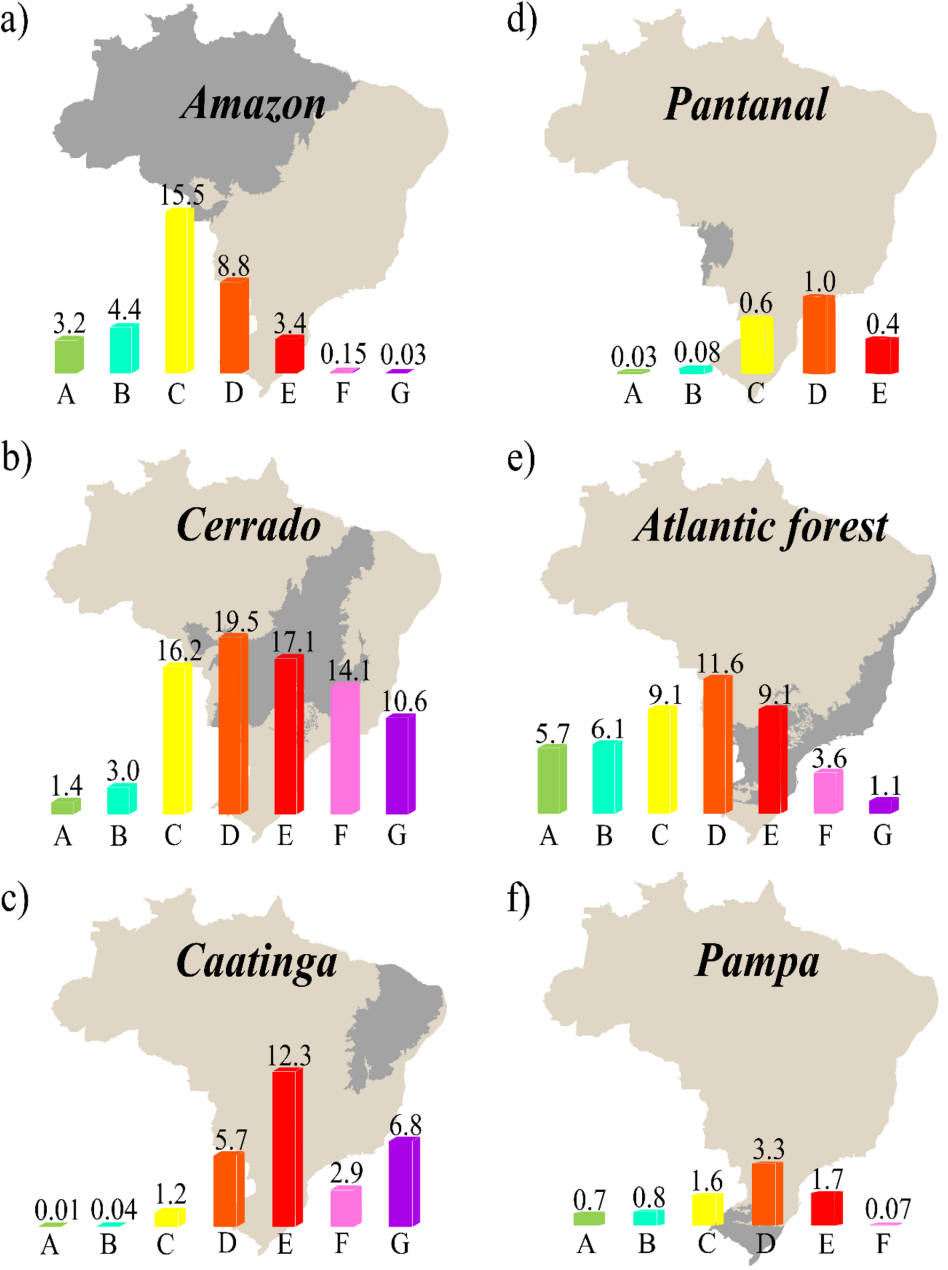
Distribution of SoilPP classes for each biome in million hectares (M ha). (**a**) Amazon (**b**) Cerrado (**c**) Caatinga (**d**) Pantanal (**e**) Atlantic Forest and (**f**) Pampa biomes. Letter A represents very high potential while letter G represents soils with very low potential. Letters between these two extremes (B, C, D, E, and F) represent high, medium/high, medium, medium/low, and low potentials, respectively. The figure was created by the software QGIS Development Team (2021). Version 3.22. http://qgis.osgeo.org and Inkscape Project. (2021). Version 1.1. https://inkscape.org.

In the Amazon biome, 35.5 M ha (17.5%) are used for agriculture (Fig. 2a). The most agricultural soils in the Amazon biome had SoilPP ranging from very high to medium. The very high, high, medium/high, and medium SoilPP correspond to areas of 3.2, 4.4, 15.5, and 8.8 M ha, respectively. About 10% of agricultural land in the Amazon had a low SoilPP (medium/low, low, and very low). About 5.5 M ha (15.5%) of the agricultural areas of the Amazon biome is occupied by soybean crop (Table S1). The Cerrado biome is the largest territorial use for agricultural activities in Brazil, occupying around 81.9 M ha (Fig. 2b). Most agricultural soils located in the Cerrado showed a medium/high to medium/low SoilPP with an area of 52.8 M ha (65% of the biome). Low and very low SoilPP in the Cerrado biome represents an area of 24.7 M ha, while high and very high SoilPP are distributed in 4.4 M ha. The Cerrado is the biome with the largest occupation of its agricultural area with soybean crop, with about 18.5 M ha (22.5%), and the second largest for sugarcane, occupying 2.8 M ha (3.5%) (Table S1).

The Caatinga biome had an estimated 29 M ha occupied for agricultural use (Fig. 2c). Most of the agricultural soils in the Caatinga biome show a medium to very low SoilPP (96% of the agricultural soils in the Caatinga). The Pantanal biome had most agricultural soils ranging from medium/high to medium/low classes, with 2 M ha (Fig. 2d). The Atlantic Forest (Fig. 2e) is the second-largest Brazilian biome in terms of land use for agricultural activities, occupying an area of about 46.3 M ha. Most agricultural soils located in the Atlantic Forest had a SoilPP ranging from very high to medium (70% of the Atlantic Forest territory). Soils with low and very low SoilPP are distributed in 4.7 M ha (10%). The Atlantic Forest is the second biome with the largest territorial occupation by soybean crop, with about 10.2 M ha (22%), behind the Cerrado. However, it presents the largest area occupied by sugarcane, with about 5.8 M ha (12.5%) (Table S1). Finally, the Pampa biome (Fig. 2f) has most of its soils with medium SoilPP (3.3 M ha); however, a significant part of the area has soils with very high to medium/high potential (38% of total agricultural land in the Pampa). About 3.9 M ha (48%) of the Pampa is occupied by soybean cultivation.

### SoilPP and historical yield data

An analysis of the average municipal yield level for soy and sugarcane was carried out, using average yields of five crops (2016/17–2020/21) collected from the SIDRA platform, where statistics by country of agricultural products can be found (https://sidra.ibge.gov.br/home/lspa/brasil)^4^. The historical yield data were classified into seven groups for both crops to be able to compare with the SoilPP classes. After that, we subtracted the productive potential of the soil from the average municipal yield level for the respective crops, which resulted in three different results: (i) positive values, meaning that the real yield of the county is above the potential of the soil, i.e., on average the crops are well managed in the county in question; (ii) result equal to 0, means that the average municipal yield level is equivalent to the productive potential of the soil, i.e., real and potential state are equal; (iii) negative values, it means that the average municipal yield (actual) is below the potential of the soil, i.e., the average yield of the culture can be increased in the county, which is possibly facing a problem in the productive chain of the culture.

**Figure 3:**
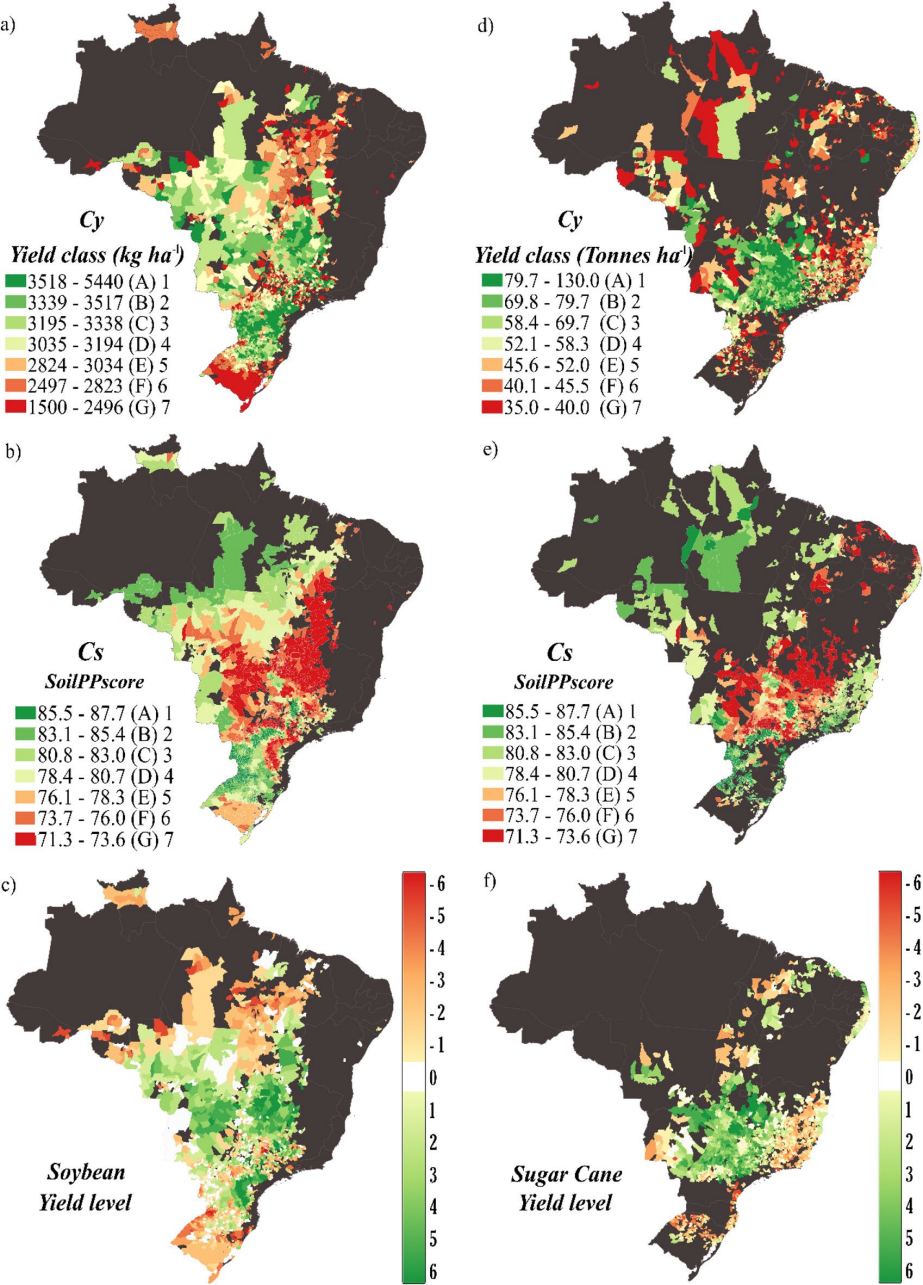
Yield class, SoilPP score, and Yield level for soybean (**a**–**c**) and sugarcane (**d**–**f**) at the municipal level. The figure was created by the software QGIS Development Team (2021). Version 3.22. http://qgis.osgeo.org and Inkscape Project. (2021). Version 1.1. https://inkscape.org.

In total, 2304 Brazilian counties with average soybean yield above 1500 kg ha^−1^ (Fig. 3a) were analyzed. After subtracting the historical yield level from the SoilPP we found that for soybean 896 counties showed positive values (Fig. 3c). Only a few counties (356) had a yield level equal to SoilPP, representing yield levels where soybean crops are at middle levels, but their yields can be increased in these counties. Low yield levels were observed in 1052 counties, which are represented by negative values in Fig. 3c. For sugarcane, 2119 counties with average yield above 35 tonnes ha^−1^ (Fig. 3d) were analyzed. 1014 sugarcane-producing counties showed positive yield level values, that is, the yield of sugarcane plantations in these counties is above SoilPP (Fig. 3f). On the other hand, 296 counties had a yield level equal to SoilPP, that is, the yield level of sugarcane crops is at adequate levels, but their yield can still be increased in these counties (Fig. 3f). Finally, 809 counties presented sugarcane yield below its potential, meaning that it can be improved by management.

### SoilPP vs. Production Environment System (PES) for sugarcane

**Figure 4:**
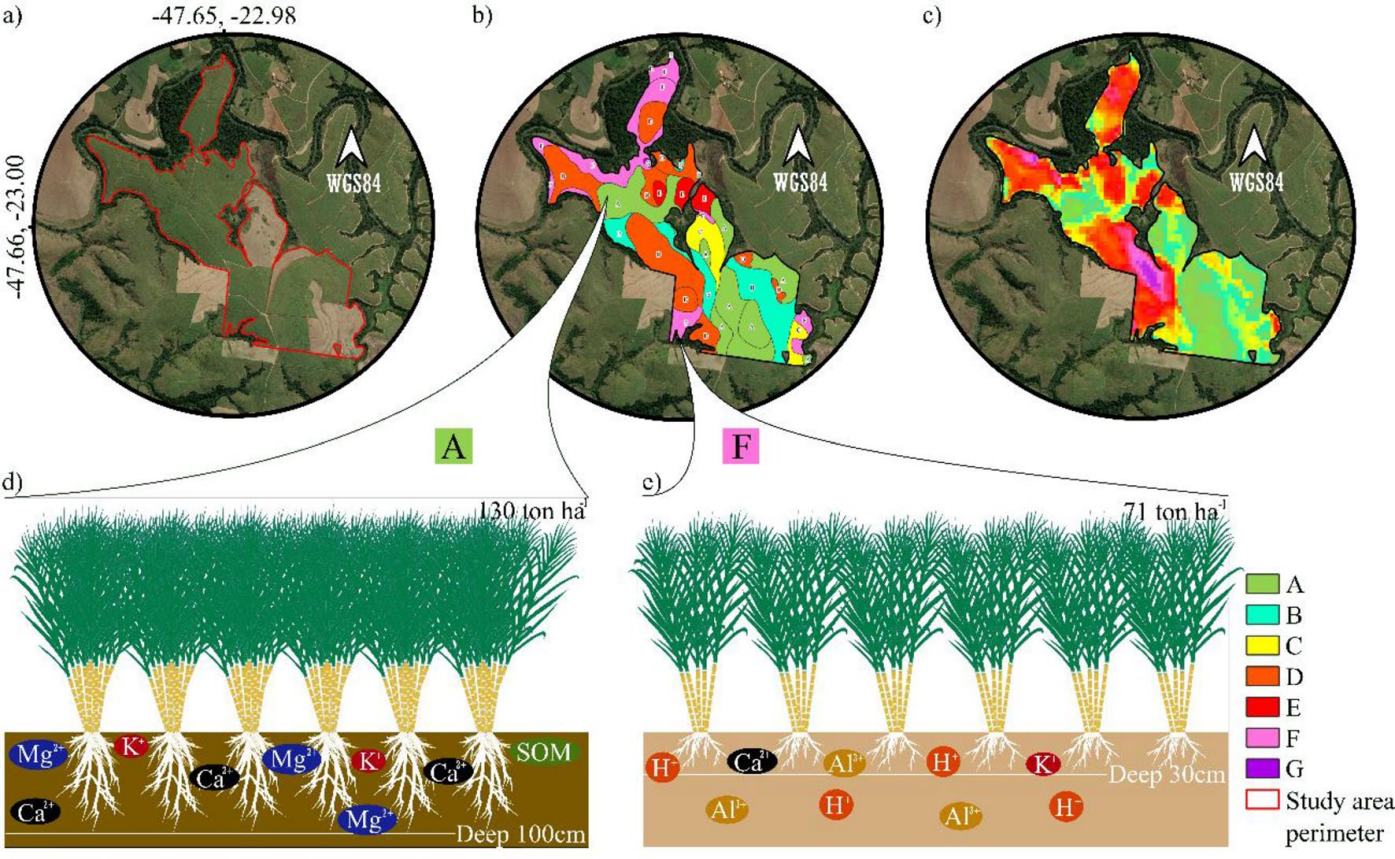
**(a**) Limit of the study area (Rafard Farm); (**b**) Empirical production environment for sugarcane; (**c**) SoilPP map. (**d**) Yield of sugarcane in environment A. (**e**) Yield of sugarcane in environment F. Sugarcane yield categories: A: greater than 100 tons ha^−1^; B: 90–100 tons ha^−1^; C:86–90 tons ha^−1^; D: 81–85 tons ha^−1^; E: 76–80 tons ha^−1^; F: 71–75 tons ha^−1^; G: less than 70 tons ha^−1^. The figure was created by the software QGIS Development Team (2021). Version 3.22. http://qgis.osgeo.org and Inkscape Project. (2021). Version 1.1. https://inkscape.org.

In order to validate our product, a comparative analysis was carried out between the SoilPP map and the Production Environment System (PES) at the farm level. The PES was developed after some empirical studies initiated by Copersucar^16^ and ratified by Landell et al.^12^, who observed a strong relationship between soil types and sugarcane yield. The authors emphasis that a soil class brings the horizon B characteristics, which is the most position that impacts the soil potential productivity. The basis (pilars) of this system are the soil type (classification), texture, fertility, depth, water retention (from surface to subsoil, 1 m), and climate. The PES is categorized into seven different classes (Fig. 4), representing the potential of soil to produce sugarcane (in tonnes ha^−1^) and represented by a specific color.

The area has 182 ha (located in the city of Rafard, São Paulo state) (Fig. 4a). Figure 4b presents the PES for the sugarcane farm. Figure 4d exemplifies soil conditions for environment 'A' which has a higher production of plant biomass where the soil has a clayey texture and base saturation (V%) above 50%. Figure 4e demonstrates the soil and plant conditions of an 'F' environment, where this soil has a very sandy texture, base saturation below 50%, and the presence of Al^3+^. The SoilPP map is represented in Fig. 4c, making it possible to compare its similarities and differences with the PES (Fig. 4b) for the sugarcane crop. The correspondence between areas with the highest SoilPP (A) and PES mapping units with the highest sugarcane yields (A) (Fig. 4b and c) is remarkable, i.e., the most productive environments classified in the field present similarities when related to the best SoilPP classes.

### Correspondence analysis between SoilPP vs. Production Environment System

**Figure 5:**
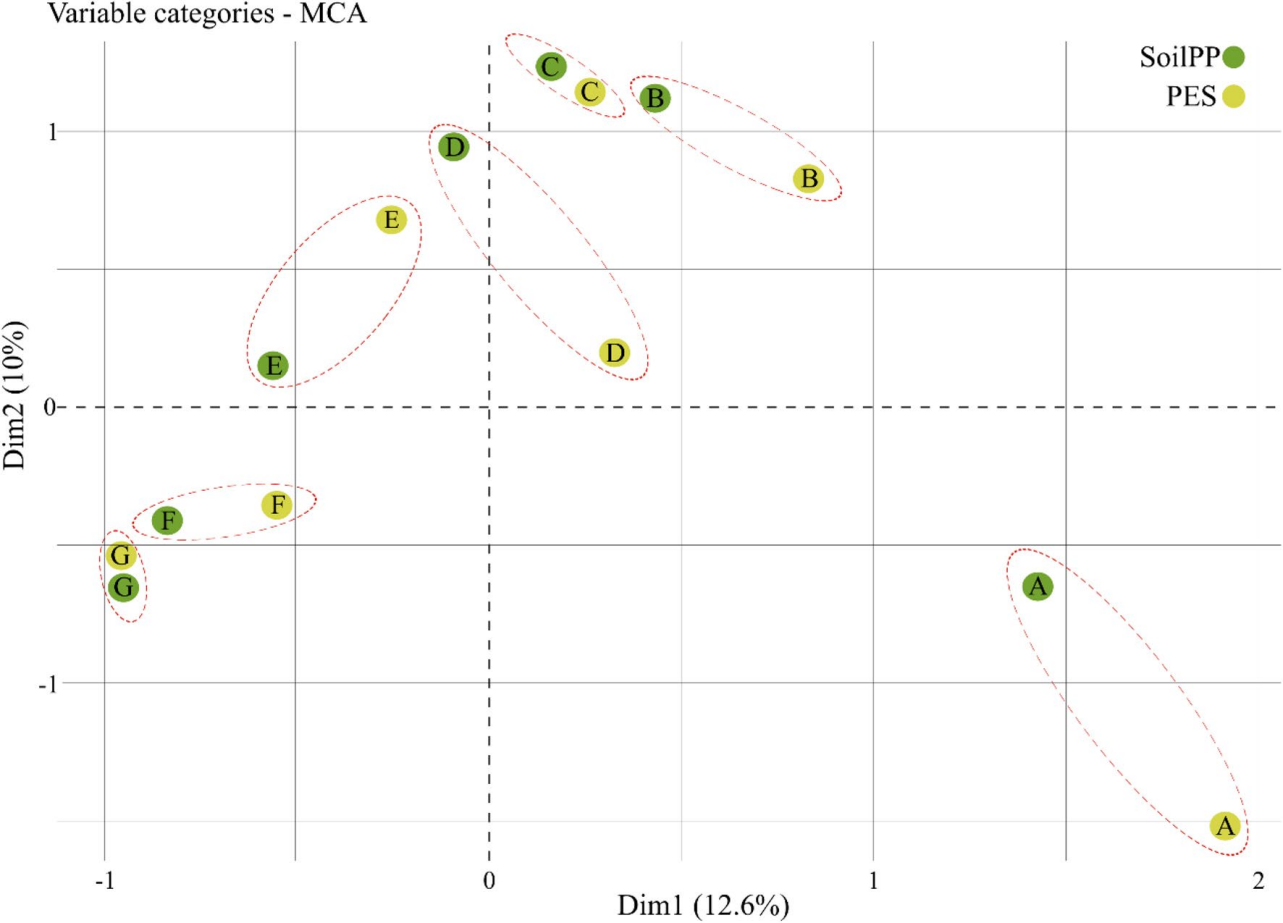
Correspondence analysis between Production Environment System (PES) and SoilPP. The figure was created by the software R Core Team (2021). Version 4.1.2. https://cran.r-project.org/bin/windows/base/old/4.1.2/

Multiple Correspondence Analysis (MCA) was performed with PES against SoilPP, to verify the association of categorical groups between both systems. The spatial association between SoilPP and PES classes^12–16^ is confirmed by correspondence analysis (Fig. 5). The soil samples that obtained the highest scores (class A) via soil productive potential index, express a significant association with class A via “PES” determined in the field. Therefore, the soils that had the highest productive potential show high similarity with the best categories of production environments. Soil samples that had the lowest SoilPP scores (classes F and G) presented greater correspondence with the worst classes of PES (Classes F and G) evaluated in the field. Category "D" expressed low correspondence between the two methods (Fig. 5). Categories B and C showed good correspondence between the different methods. Therefore, it is notable that the numerical system that evaluates SoilPP through the scoring of soil properties corresponds to an empirical method of PES determined in the field.

## Discussion

### SoilPP for Brazilian agricultural areas

Brazilian agricultural soils that have the highest SoilPP (Class A, B, and C) are located in areas of source material from volcanic rocks, such as basalt (Figs. S1 and S2)^31^. Basalt is recognized for developing productive soils^17^, forming soils with good natural fertility due to the availability of nutrients (i.e., including P, K, Ca, Mg, and Fe) and smaller granulometry, which favors the storage and availability of water for the plants^10^. Consequently, Brazilian soils with very high and high SoilPP are in areas of high soil fertility, in agreement with the results found by Embrapa^32^, which represented the fertility of Brazilian soils. The textural gradient is the ratio of the percentage of clay on the subsurface horizon compared to that of the surface horizon^19^. This characteristic is usually present in Argissolos/Lixisols, which is the second most occur soil in Brazil's agricultural areas (Fig. S1b). These soils have greater water availability due to their textural difference, increasing the residence time of water in the soil profile^19^. When roots are in undersurface, they have more chances to absorb water and nutrients and thus increasing productivity.

In addition, areas of agricultural soils with very high and high SoilPP have the highest concentrations of SOC (Fig. S2). In agreement with Huang et al.^11^, SOC has the potential to improve soil structure (such as aggregate stability) and retention of water and nutrients (such as nitrogen) in soils^11^. Other studies indicate that increasing SOC stock can increase soil quality (improvement in the performance of certain soil functions, such as water retention, aggregation, and cation exchange capacity) and improve crop yields^33–35^. This is in agreement with our findings. The territories with the highest concentration of SOC in Brazilian agricultural areas^36^ correspond with the highest values of SoilPP since SOC has a direct influence on the production of biomass of cultivated plants^11^.

It can encourage farmers to improve the management to increase soil organic matter (SOM) content, intensifying production systems, such as by crop rotation and diversification (e.g., grasses and legumes), which are efficient strategies to increase soil C contents^37–39^. In other words, the agricultural contribution can occur with global initiatives such as “Soil Carbon 4 per Mille”, which proposed to increase SOC sequestration, mitigating the harmful effects of global anthropogenic greenhouse gas emissions^40^. In order to increase global food security for a rapidly growing population under a changing climate, best management practices should be adopted to improve soil structure and SOC stocks to improve water storage, nutrient retention, and energy conservation^11^.

The highest concentration of agricultural areas with SoilPP (medium/high to medium/low) is mainly located in the Cerrado (52.8 M ha) and Atlantic Forest (29.8 Mh ha) biomes (Fig. 1). Areas with SoilPP (low to very low) are located mainly in the Caatinga (9.7 M ha) and Cerrado (24.7 M ha) biomes. According to MapBiomas^5^, the Cerrado biome has a pasture area equivalent to 47 M ha, where 57.9% (27 M ha) are in severe or moderate levels of degradation. The Caatinga biome has about 18 M ha occupied by pastures^5^. However, 70.3% (12.6 M ha) of this culture are in severe or moderate levels of degradation. Therefore, these areas of degraded pastures may be related to the areas of low to very low SoilPP, due to chemical and physical soil limitations.

Areas with low to very low SoilPP in the Cerrado have low base saturation, low pH, and high aluminum concentration^32^, which together with the large amount of Fe and Al oxides (highly weathered soils)^19,41,42^, generate phosphorus restriction problems. Acidification of agricultural soils is a worldwide problem and reversing it improves nutrient uptake, root growth, and crop yields^17,43^. Therefore, the potential of these areas can be increased through management, such as neutralization of acidic soils (liming), reducing metal toxicity (e.g., aluminum and manganese levels) and increasing P availability, especially in highly weathered tropical acidic soils where metal oxides bind strongly to remaining P reserves^17^. These factors directly affect the soil's capacity to produce biomass. It is necessary to emphasize, that these characteristics usually occurs in all profile (0-100 cm). Although, the Brazilian soil agriculture community has worked hard in chemical management in the last 30 years at the soil surface. With this, most of Cerrado agricultural soil has been corrected (at superficial layers) and productivity improved. The increase in productivity by soil chemistry management is a fact, but the topic here is to say that it did not reach the maximum of its potential. This is true when we see the fundamental of SoilPP and the 30 years of experience in eucalyptus and sugar cane using PES, that reside on soil analysis in the undersurface (80-100 cm). It is until this depth that roots have more chances to absorb water and nutrients coming from soil drainage, as preconized by^12^.

The best agricultural soils in Brazil due to their territorial extension that belong to the SoilPP categories “very high and high” are found in the Atlantic Forest (11.8 M ha), Amazon (7.6 M ha), and Cerrado (4, 4 M ha) (Fig. 2a, b, and e). These three biomes together account for about 81% of the assessed agricultural area, being the most expressive in soybean and sugarcane production in terms of quantity (Table S1). 13,5% of the Amazon biome is occupied by agriculture. However, soils located in the Amazon are extremely fragile in chemical and physical terms, since the cycling of organic matter is one of the main factors that help in the availability of nutrients^44,45^. Another limitation is the occurrence of flooded areas due to the high rainfall that occurs in the biome^46^. In addition, Federal law No. 12.651/2012, which provides for the protection of native vegetation, requires that all rural properties located in the Amazon maintain 80% of the area covered by native vegetation^44^.

### SoilPP vs. national agricultural yield data

Through the yield level analysis of 2304 Brazilian soy-producing counties, around 1052 counties were identified that could increase their production levels (Fig. 3c). For sugarcane, 2119 producing counties were evaluated, where 809 counties could increase crop yield (Fig. 3f). Some Brazilian counties had high average yields for both crops even at low SoilPP values, such as the Southeast, Midwest, and Northeast regions (Fig. 3a, b, d, and e). This is possibly associated with the level of technological investment of the counties in the respective regions^47^. For example, the regions with the adoption of irrigation practices have the highest average yield per county, which can be verified through the map of irrigated Brazilian agricultural areas (National Water and Sanitation Agency^48^). Another factor is the large Brazilian reservoirs of residual phosphorus (P) accumulated over decades of cultivation, called the “P legacy”. Pavinato et al.^49^ demonstrated this, by mapping the spatiotemporal distribution of the P legacy in the last 50 years in Brazil. Some regions with the highest accumulation of P legacy in Brazilian soil (greater than 300 kg ha^−1^) demonstrate the intense technological investment in counties with low SoilPP to leverage production levels for soybeans and sugarcane^49^.

The highest values of Gross Domestic Product “GDP” per capita in 2019^50^ correspond to regions of irrigated areas that belong to some agricultural frontiers of strong expansion in Brazil^48^. High yield might be associated with a high technological investment in these regions, such as fertilization^49^ and irrigation^48^. Thus, the development of public policies to increase technological investment in counties with a negative Yield level (Fig. 3c and f) could result in an increase in their average yield without the exploitation of new agricultural areas, contributing to food security^11,22^. In agreement with Strassburg et al.^45^, “Brazil has enough land under agricultural production to meet the increase in future demand for agricultural products, saving land for nature, i.e., combining increased agricultural yield with the conservation and restoration of natural environments”. Thus, meeting the food security and sustainability challenges of the coming decades is possible, but will require considerable changes in nutrient and water management^47^.

### SoilPP vs. Production Environment System (PES) for sugarcane

Sugarcane, cultivated extensively in highly weathered soils, generates approximately US$43 billion per year for the Brazilian economy^17^. Production environments with higher sugarcane yields (Fig. S3) have deeper, fine-textured soils and higher soil fertility^19,51,52^. Subsurface soil information directly impacts crop production due to its root exploration for water and nutrients, which justifies why the productive potential of the soil is greater in production environments with the highest yields^18^. The worst soils in the area classified by PES and SoilPP map have a coarse texture (sandy) and are chemically poor^51^. Soil acidification reduces nutrient uptake, root growth, and crop yields^17,43^. The neutralization of acidic soils would be an efficient alternative to improve the exploitation of the soil's productive potential^17^. There is a straight relationship between SoilPP and where both uses of undersurface (at least 1 m) soil attributes information. The difference is that PES use a soil map classification and SoilPP can use soil analysis directly. Despite this, SoilPP integrated carbon into the system differently than PES. In both cases, it was proven that the undersurface analysis gives an important inference to soil types and impacts directly on the potential productivity.

### Contributions and limitations of SoilPP for understanding agricultural soils in Brazil

Legacy soil maps strongly supported Brazilian agricultural expansion^53^. However, soil maps in Brazil date from the 70 s and 80 s “RadamBrasil” and have low cartographic detail (1:500.000 to 1:5.000.000). Nevertheless, despite the low resolution of these soil maps, they greatly contributed to agricultural development in several areas of Brazil, promoting more efficient and sustainable use of the land^36^. Currently, there is great interest in the elaboration of detailed maps, promoting more efficient and sustainable use of land^36^. These techniques can help increase global food security^11,22^, through the aid of national-scale policy formulations, such as “Brazil’s National Soil Program”—PronaSolos^53^ and Iowa Corn Suitability^20^. They can also contribute to agricultural resource management and soil security^21–23^.

The main limitations of our product (SoilPP map) (Fig. 1) are related to the low accuracy of the forecast model and to agricultural areas without information on exposed soil reflectance, which allowed us to assess 80% (203 M ha) of the total Brazilian agricultural areas (263 M ha)^5^. Techniques for soil management and plant nutrition, such as limestone and fertilizer application, no-tillage, and biological nitrogen fixation, justify the high dynamics of soil chemical attributes^17^. The low accuracy of the prediction model is possibly associated with the evaluation of chemical attributes in SoilPP, which was observed in predictions of chemical attributes by machine learning^36,42,54,55^. Despite the complexity of working with the mapping of large territories, the importance of evaluating the potential of Brazilian agricultural soils is remarkable, providing knowledge related to soil limitations in different places, and aiming at increasing agricultural yield sustainably.

## Final comments

The method presented in this work based on soil properties (indicators) associated with digital soil mapping represented the productive potential of agricultural soils in Brazil. The SoilPP map presented the following limitations: (1) the sample representativeness for all types of agricultural soils; (2) a low accuracy of the prediction model, possibly related to the high variation of chemistry and (c) was not able to make one script for each culture. Despite this, about 203 Mha (80% of the total agricultural areas) were mapped with soil information up to 1 m deep and a spatial resolution of 30 m.

The system was analyzed using empirical models and real yield data with good correspondence. 46% of Brazilian counties with soybean cultivation could increase yield. For sugarcane, 38% of the evaluated counties had real average yield below the soil potential. Therefore, for some counties, the amount of sugarcane and soybean produced could be increased where the crops are already grown (Yield Gap) if their average municipal yield were increased (Tables S2 and S3). The results support the importance of undersurface soil data in the impact of production. The community should give more importance to undersurface soil information since the more roots, the greater absorption of nutrients and water. Until now, agriculture has given too much attention to surface conditions which rimel the real potential of soil productivity. For this, attention has to be given to soil survey (soil pedological mapping), which brings to light the soil drainage dynamics and the undersurface condition. Furthermore, the system indicated that 13.5% of the Amazon biome is already under agriculture. Therefore, once the data set was elaborated, important information was generated on a fine scale, with a relevant approach to several areas such as public policies, knowing the purpose of certain land, land prices, optimization of soil management, areas chosen for the management of crops, and increase yield. This will affect the reduction of land degradation, mitigate deforestation and contribute to global food security.

## Methods

The sequence to obtain the SoilPP was carried out in four main steps. The organization of the national soil database (Step 1). The scoring process of each soil sample by Principal Component Analysis (Step 2). The prediction SoilPP map for the entire territory using the DSM framework (Step 3). Finally, the SoilPP evaluation using real sugarcane and soybean yields at the county level and production environments at the farm level (Step 4). The selection of sugarcane and soybean crops to validate this product responds to two main reasons: (i) the importance of these two crops to the Brazilian economy^4^ and (ii) their widespread distribution across the country.

### Study site

The study area involves all agricultural lands from Brazil. The Collection 6.0 from the MapBiomas program was used to select the areas in which agriculture was the main land use, resulting in 263,045,118 M ha^5^. The Brazilian territorial extension shows a wide biodiversity, with six biomes and different climatic conditions^26,56^. The main soil types are derived from sedimentary and igneous rocks, such as Ferralsols and Acrisols, covering about 60% of the Brazilian territory^19^. The Amazon biome represents 49% of Brazil, with a humid tropical climate (Af, Am, and Aw). The Cerrado (Brazilian savannas) is the second largest biome, covering 22% of the territory, with a predominantly semi-humid climate (Aw). The Atlantic Forest biome, which extends to the east of Brazil, has the greatest diversity of environments and there are several types of climates (Cfb, Cfa, Cwb, Aw, and As). The Caatinga biome is the driest, under a semi-arid climate (Bsh). The Pantanal biome is characterized by long periods of flooding, with seasonal climate classified as Aw. The pampa biome is covered by temperate grasslands with a Cfa climate.

### Soil database

The georeferenced soil samples were selected from soil surveys carried out in agricultural areas by the Geotechnologies in Soil Science group^57^ from the University of São Paulo. All sampling points at three depth layers (layer A: 22,122; layer B: 21,160; layer C: 25,992) had their physical and chemical soil attributes analyzed. The physical–chemical laboratory analysis followed Brazilian standards^32^. The following soil attributes were obtained: clay, silt, and sand (g kg^−1^) contents; soil organic carbon (SOC, g kg^−1^); soil organic matter (SOM, g kg^−1^); pH in water (pH_H2O_); pH in KCl (pH_KCl_); cation exchange capacity (CEC pH7); Ca^2+^ (mmol_c_ kg^−1^); Mg^2+^ (mmol_c_ kg^−1^); K^+^ (mmol_c_ kg^−1^); Al^3+^ (mmol_c_ kg^−1^); H + Al (mmol_c_ kg^−1^) sum of bases (mmol_c_ kg^−1^); base saturation (V%); and aluminum saturation (m%). Soil bulk density (BD, g cm^−3^) was estimated using a pedotransfer function for Brazilian soils calibrated with clay and SOC contents, which had an R^2^ value of 0.63 and a standard error of 0.11 g cm^−3^^58^. The ∆pH, soil weathering index (Ki), and clay activity were calculated according to Prado et al.^59^. Additionally, the slope (º) of the surface was calculated from the ALOS Landform dataset, in the Google Earth Engine platform using the TAGEE function^36^. Relief is a factor in soil formation, in which the greater slopes of the terrain present shallower soils and are susceptible to erosion^60^. Information on terrain slope from the raster file was extracted for each geo-referenced soil sample point.

### Building the soil productive potential scoring index (SoilPP)

The soil potential yield index (SoilPP) was developed considering the soil quality literature and indexing methods^21,29^. To build the SoilPP, soil properties were scored with values ranging from 0 to 100. The scoring functions are divided into three types: ‘more is better’ index (MBI), which means that higher values of the soil attribute indicate higher SoilPP; ‘less is better’ index (LBI), in which lower values indicate high SoilPP; and “optimal midpoint” (IMO), where an intermediate value indicates superior soil condition^29^. For Clay, Silt, SOC, SOM, Ca^2+^, Mg^2+^, K^+^, CECpH7, SB, V%, Ki, and Clay activity the MBI was used (Eq. 1). Sand, BD, Al^3+^, H^+^ + Al^3+^, m%, ∆pH, and Slope were scored using LBI (Eq. 2), and only pH_H2O_ was scored using OMI (Eq. 3).

1 $$MBI= \frac{a}{\left[1+{\left(\frac{B-UL}{x-UL}\right)}^{S}\right]}$$

2 $$LBI=\frac{a}{\left[1+{\left(\frac{B-LL}{x-LL}\right)}^{S}\right]}$$

3 $$OMI=\begin{array}{ccc}\frac{a}{\left[100-{\left(\frac{{B}_{L}-O}{x-O}\right)}^{S}\right]}& for& x<O\\ 100& for & x=O\\ \frac{a}{\left[100-{\left(\frac{{B}_{U}-O}{x-O}\right)}^{S}\right]}& for & x>O\end{array}$$

where MBI_(1)_, LBI_(2),_ and OMI_(3)_ are the 'more is better', 'less is better', and 'optimal midpoint' scoring functions, respectively; ‘*a*’ is the maximum score value (100), ‘*S*’ is the slope of the equation, defined as − 2.5; ‘*B*’ is the baseline value that has a score of 50% (median); ‘*U*_*L*_’ is the upper (maximum) limit of the soil attribute value; ‘*L*_*L*_’ is the lower (minimum) limit of the soil attribute values; ‘*B*_*L*_’ is the lower baseline of the 'ideal midpoint' curve, with a score of 50%; ‘*B*_*U*_’ is the upper baseline of the 'ideal midpoint' curve, with a score of 50%; ‘*O’* is the optimal score value, equal to 100%; and ‘*x’* is the real value of the soil attribute.

Each soil attribute was parameterized with baselines (median), limits (upper and lower), and optimal values through statistical reductions (percentiles) of soil samples (Table 1). Subsequently, the attributes of each soil sample were evaluated and scored. This approach was used to restrict the function parameters to soil attributes obtained through soil analysis.

**Table 1:** Parameters of the scoring functions of soil attributes.

Attribute	Scoring function^a^	LL^b^	B^c^	UL^d^	B_L_^e^	O^f^	B_U_^g^
Sand (g kg^−1^)	LBI	9.00	747.00	974.00	–	–	–
Silt (g kg^−1^)	MBI	0.00	63.00	846.00	–	–	–
Clay (g kg^−1^)	MBI	5.00	180.00	960.00	–	–	–
Bulk Density (g cm^−3^)	LBI	0.85	1.42	1.55	–	–	–
SOC (g kg^−1^)	MBI	0.01	5.50	59.16	–	–	–
SOM (g kg^−1^)	MBI	1.00	9.40	102.00	–	–	–
pH_H2O_	OMI	3.70	5.50	7.80	5.4	6.5	7.2
Ca^2+^ (mmolc kg^−1^)	MBI	0.00	8.01	99.60	–	–	–
Mg^2+^ (mmolc kg^−1^)	MBI	0.00	3.70	55.00	–	–	–
K^+^ (mmolc kg^−1^)	MBI	0.00	0.70	20.00	–	–	–
Al^3+^ (mmolc kg^−1^)	LBI	0.00	1.50	156.50	–	–	–
H^+^ + Al^3+^	LBI	0.10	19.20	336.50	–	–	–
CEC (mmolc kg^−1^)	MBI	3.29	35.40	349.50	–	–	–
SB (mmolc kg^−1^)	MBI	0.00	13.10	159.80	–	–	–
V (%)	MBI	0.00	39.60	99.84	–	–	–
m (%)	LBI	0.00	10.58	100.00	–	–	–
∆pH	LBI	− 3.30	− 0.8	2.48	–	–	–
Ki	MBI	0.69	1.62	3.73	–	–	–
Clay activity	MBI	0.72	18.39	1887.65	–	–	–
Slope (º)	LBI	0.00	3.09	38.36	–	–	–

^a^LBI: “Less is better” index; MBI: “More is better” index; OMI: “optimum mid-point”. ^b^Lower limit. ^c^Baseline. ^d^Upper limit. ^e^Lower baseline. ^f^Optimum. ^g^Upper baseline. Obs.: The parameters were determined by statistical reductions of the whole Brazilian territory. The minimum and maximum values were determined by the 0.01 and 0.99 percentiles. The baseline was determined by the median value (0.5 percentile). The lower baseline was determined by the middle between the minimum (in parenthesis) and optimum values, while the upper baseline was determined by the optimum and maximum values (in parenthesis).

Principal component analysis (PCA) was used to create a weighted artifice index named SoilPP (Eq. 5), by evaluating all scores of soil attributes from the MBI, LBI, and OMI equations, using the methodology described by Cherubin et al.^29^. Due to the different ranges of values and measurement units of the soil attributes, their values were standardized (mean 0 and standard deviation 1) using the z-score, which can be calculated with the following formula:

4 $$Z = \frac{{x - \overline{x}}}{\sigma }$$

where *z* is the z score, *x* is the value of the soil attribute, *x̅* is the mean, and σ is the standard deviation.

Through PCA, each soil attribute was weighted according to the proportion of variance explained by each component (ie, % of variation explained by each component divided by the total accumulated variation of all components selected). The weighted values for all attributes used in the calculation of SoilPP resulting from PCA are available in the supplementary material (Tables S4 and S5). The number of principal components selected to perform the SoilPP calculation was based on the standard deviation (SD) value resulting from the principal component (PC) above 1 (Table S5). To calculate the SoilPP the following equation was used:

5 $$SoilPP=[({\sum }_{i=1}^{n}AS*WI)*PCn]$$

where *'n'* is the number of soil attributes, *'AS'* is the score obtained by each soil attribute in each soil sample, *'WI'* attribute weighted value within each main component provided through PCA, *'PCn'* is the proportion of variance of the principal component. Finally, all soil samples from each layer were scored on a scale from 0 to 100. The closer the sample score is to 100, the greater the productive potential of the soil. Principal component analysis (PCA) was performed using the “factoextra”, “lifecycle” and “psych” packages in the R environment^61^.

### Prediction of SoilPP with digital soil mapping

A Synthetic Soil Image (SySI), obtained by applying the GEOS3 method^62^ was used in conjunction with terrain attributes^63^ to estimate the SoilPP for Brazilian agricultural areas, using digital soil mapping. A set of bootstrap and random regression trees from Python's scikit-learn library were used^64^. Instead of fitting simple regression trees, we emulated the Random Forest algorithm^65^ generating bootstrapping trees to aggregate them by the mean, using the methodology described in Safanelli et al.^36^. The optimal number of bootstrap trees (forest size), number of covariates to be sampled in tree divisions and tree size was defined with a hyperparameter grid search seeking to minimize overfitting during calibration. The range of values tested for forest size was 30, 60, 100, 200, 300, and 500 trees. The amount of 1, 2, 3, 5, 8, 11, and 13 predictors was investigated to be used randomly in three divisions. For the minimum number of observations on the leaves, which defined the size or individual depth of the tree, values of 10, 20, 30, 40, 50, 100, 200, and 500 observations were tested. Detailed information is available in the supplementary material.

### Analysis of SoilPP with historical yield data (national extent)

The predicted SoilPP map was intersected with mean crop yield data of soybean and sugarcane from the Brazilian counties, averaged over five years (2016/17–2020/21). SoilPP map and the municipal average yield data for the respective crops were used to perform this assessment on a national scale. The county yield data were obtained as vector files from the Brazilian Institute of Geography and Statistics database (IBGE, https://sidra.ibge.gov.br/tabela/6957). The counties that had average production above 35 tons ha^−1^ of sugarcane and 1500 kg ha^−1^ of soybean were considered in the analysis. The average yield values of each county for soybean and sugarcane were categorized based on quartiles (Table 2), i.e., each category has the same number of counties, in order to minimize the overestimation of the increase in yield. After preparing the SoilPP map, the average of the agricultural soils that make up each Brazilian county was performed, that is, the result is an average value of the soil potential for each county, which was categorized as 'SoilPPc' (Table 2). The municipal average yield values for soybean and sugarcane crops (Table 2) are in agreement with values found in the literature^66–69^. The values of Brazilian counties with the average yield for soybean and sugarcane were divided into seven different classes, called Yield Class ‘Yc’. The variables SoilPPc and Cy are available in Table 2 and were used to calculate the level of yield.

**Table 2:** Categorization of the average yield yield for sugarcane and soybean crops with the corresponding SoilPP score and SoilPP class.

Soybean	Sugar cane		SoilPP index	
Yield (kg ha^−1^)	Yield (ton ha^−1^)	Yield class (Y_c_)	SoilPP score	SoilPP class (SoilPP_c_)
3517.0–5440.0	79.7–130.0	1	85.5–87.7	1
3339.0–3517.0	69.8–79.7	2	83.1–85.4	2
3194.0–3338.0	58.3–69.8	3	80.8–83.0	3
3034.0–3194.0	52.0–58.3	4	78.4–80.7	4
2823.0–3034.0	45.6–52.0	5	76.1–78.3	5
2496.0–2823.0	40.0–45.6	6	73.7–76.0	6
1500.0–2496.0	35.0–40.0	7	71.3–73.6	7

The SoilPPc Class score (Cs) and Yc variables are used in Eq. (6).

In order to verify the actual state of municipal yield for soybean and sugarcane crops (2016/17 to 2020/21 harvests) in relation to the average potential of agricultural soils for each Brazilian county. The analysis of the municipal yield level was performed. Through this analysis, it is possible to see if the average municipal yield of the respective crop yield can be increased in the agricultural lands where it is already cultivated, or if there is a need to open new agricultural areas to produce more. 'SoilPP_c_' and 'Y_c_' were categorized for each Brazilian county according to Table 2. 'SoilPP_c_' was subtracted from 'Y_c_' to obtain a difference map:

6 $$Yield\;level = {\text{SoilPPc}} - {\text{Yc}}$$

The equation represents the difference between the actual state (real yield) and potential state (SoilPP) of the soil to produce biomass. If the resulting value of the “Level of yield” is positive, it means that the average municipal yield (actual) is above the potential of the soil, that is, the agricultural areas cultivated with soybean or sugarcane, on average the crops are well managed in the county in question. Conversely, if the resulting value is negative, it means that the average municipal yield (actual) is below the potential of the soil, that is, the average crop yield can be increased in the county, which is possibly facing some problems during the production (climate factor, competition for weeds or attack by pests and diseases). The counties that obtained a result equal to 0, demonstrate that the actual and potential states are equal, meaning that the full potential of the soil is being explored. However, crop yields can still be increased.

### Validation of the SoilPP with production environments for sugarcane

A case study was carried out on a sugarcane farm, located in the county of Rafard (State of São Paulo). The farm's area is about 182 ha. We used this example as a basis for validation of the SoilPP map and comparison with the PES^69^. This framework consists of developing ‘management zones’ using information on soil class, texture, soil fertility, and yield. The PES has seven different categories based on sugarcane yield (A: greater than 100 tons per hectare; B: 91 to 100 tons; C: 86 to 90 tons; D: 81–85 tons, E: 76–80 tons; 70–75 tons and G: < 70 tons per hectare). SoilPP map relationships with the geology and soil classification of the area were also analyzed through legacy field maps, enabling a better understanding of the characteristics of a given environment. The SoilPP map of the Brazilian agricultural areas was cut according to the perimeter of the Rafard farm (São Paulo State), presenting a spatial resolution of 30 m and soil information up to a depth of one meter. The PES was generated from the physical, chemical, and biological properties of the soil, plant evapotranspiration, and yield, which were evaluated in the field.

Multiple Correspondence Analysis (MCA) was performed using 21,000 soil samples evaluated in the field via PES and used in SoilPP map prediction, to verify the association of categorical groups between both systems. The variables analyzed were the SoilPP score compared to PES, which is categorized through pedological classification, with information on texture, fertility, and evapotranspiration of the plant^69^. The PES is divided into seven different categories (A, B, C, D, E, F, and G) based on sugarcane yield, as described in the previous item. With this, it is possible to analyze whether the SoilPP categorical system (Eq. 5) corresponds with the empirical categorical system performed through the methodology of Demattê and Demattê^69^. The Chi-square test was applied to assess the significance of the association between the categories of both methods.

### Supplementary Information

Supplementary Information.

## Data Availability

The data generated and/or analyzed during the current study are available in the repository (https://esalqgeocis.wixsite.com/english/lucas-greschuk-soil-pp-map). Some data used and/or analyzed during the current study may be made available by the corresponding author upon request via e-mail jamdemat@usp.br. This research was developed based on real field information and municipal yield.
