# Supplementary material for: A soil productivity system reveals most Brazilian agricultural lands are below their maximum potential
Source: Sci Rep. 2023 Aug 29;13:14103. doi: 10.1038/s41598-023-39981-y (PMC10465562; doi:10.1038/s41598-023-39981-y)
Supplement: Supplementary file 1 — Supplementary Information. [file 41598_2023_39981_MOESM1_ESM.docx]

**Supplementary information**

**Table S1:** Percentage of each class of soil productive potential in each biome and the area occupied by soybean and sugarcane.

|  | **SoilPP (%)** | | | | | | |  | **MapBiomas (2021)** | | | |
| --- | --- | --- | --- | --- | --- | --- | --- | --- | --- | --- | --- | --- |
|  | **A** | **B** | **C** | **D** | **E** | **F** | **G** | **agriculture area (%)** | *Sugarcane area (%)* | *Soybean area (%)* | *Sugarcane area (ha)* | *Soybean area (ha)* |
| **Amazon** | 9.02 | 12.40 | 43.69 | 24.80 | 9.58 | 0.42 | 0.08 | 17.49 | 0.20 | 15.33 | 76353 | 5439820 |
| **Pantanal** | 1.42 | 3.79 | 28.44 | 47.39 | 18.96 | 0.00 | 0.00 | 1.04 | 0.00 | 0.15 | 160 | 3192 |
| **Cerrado** | 1.71 | 3.66 | 19.78 | 23.81 | 20.88 | 17.22 | 12.94 | 40.36 | 3.41 | 22.55 | 2786324 | 18466298 |
| **Atlantic forest** | 2.81 | 3.01 | 4.48 | 5.72 | 4.48 | 1.77 | 0.54 | 22.82 | 12.47 | 22.09 | 5771724 | 10226301 |
| **Caatinga** | 0.00 | 0.02 | 0.59 | 2.81 | 6.06 | 1.43 | 3.35 | 14.27 | 0.15 | 0.03 | 43233 | 8433 |
| **Pampa** | 0.34 | 0.39 | 0.79 | 1.63 | 0.84 | 0.03 | 0.00 | 4.03 | 0.00 | 47.61 | 0 | 3889586 |
| **Brazil total** | 5.44 | 7.11 | 21.78 | 24.59 | 21.68 | 10.26 | 9.13 | 100.00 | 4.28 | 18.74 | 8677802 | 38033646 |

*%: percentage; ha: hectare.

**Table S2:** Sugarcane production parameters for some Brazilian municipalities.

| **City code** | **City name** | **State** | **City area km^2^** | **Sugarcane yield (ton/ha)** | **Sugarcane area (ha)** | **Amount produced (ton)** | **Attainable production (ton)** | **Yield GAP (ton)** |
| --- | --- | --- | --- | --- | --- | --- | --- | --- |
| *cities with a high average yield* | | | | | | | | |
| 5211909 | Jataí | GO | 7174.219 | 120.00 | 16646 | 1997520.0 | 1997520 | 0 |
| 3508504 | Caçapava | SP | 368.99 | 116.00 | 183 | 21228.0 | 21960 | -732.0 |
| 3555802 | Urânia | SP | 209.262 | 116.00 | 3198 | 370968.0 | 383760 | -12792.0 |
| 3556404 | Vargem Grande do Sul | SP | 267.178 | 105.00 | 4967 | 521530.0 | 596040 | -74510.0 |
| 3512407 | Cordeirópolis | SP | 137.579 | 103.67 | 6392 | 662686.8 | 767040 | -104353.2 |
| 3531803 | Monte Mor | SP | 240.566 | 102.88 | 4345 | 447003.2 | 521400 | -74396.8 |
| 3135050 | Jaíba | MG | 2635.467 | 101.87 | 7787 | 793247.7 | 934440 | -141192.3 |
| 3555901 | Uru | SP | 146.901 | 100.00 | 4746 | 474612.3 | 569520 | -94907.7 |
|  |  |  |  |  |  |  |  |  |
| *cities with lower average yield* | | | | | | | | |
| 4102109 | Astorga | PR | 434.792 | 65.16 | 2049 | 133521.4 | 245880 | -112358.6 |
| 3538709 | Piracicaba | SP | 1378.069 | 65.40 | 37262 | 2436934.8 | 4471440 | -2034505.2 |
| 3550407 | São Pedro | SP | 611.278 | 65.40 | 9378 | 613321.2 | 1125360 | -512038.8 |
| 4128302 | Uniflor | PR | 94.819 | 65.48 | 1902 | 124535.4 | 228240 | -103704.6 |
| 4123956 | Santa Mônica | PR | 259.957 | 65.52 | 3575 | 234238.3 | 429000 | -194761.7 |
| 3545159 | Saltinho | SP | 99.738 | 65.53 | 2700 | 176940.2 | 324000 | -147059.8 |
| 3547007 | Santa Maria da Serra | SP | 252.621 | 65.53 | 4299 | 281728.1 | 515880 | -234151.9 |
| 3509809 | Campos Novos Paulista | SP | 484.199 | 65.59 | 4189 | 274764.9 | 502680 | -227915.1 |
| * Sugarcane yield average (ton/ha) from 2015/16 to 2020/21 | | | | | |  |  |  |

**Table S3:** Soybean production parameters for some Brazilian municipalities.

| **City code** | **City name** | **State** | **City area km^2^** | **Soybean yield (kg/ha)** | **Soybean area (ha)** | **Amount produced (kg)** | **Attainable production (kg)** | **Yield GAP (kg)** |
| --- | --- | --- | --- | --- | --- | --- | --- | --- |
| *cities with a high average yield* | | | | | | | | |
| 3550209 | São Miguel Arcanjo | SP | 930.339 | 4800 | 17194 | 82531200 | 82531200 | 0 |
| 3510203 | Capão Bonito | SP | 1640.23 | 4500 | 28843 | 129793500 | 138446400 | -8652900 |
| 3509452 | Campina do Monte Alegre | SP | 184.479 | 4200 | 5552 | 23318400 | 26649600 | -3331200 |
| 3523503 | Itatinga | SP | 979.817 | 4163.8 | 4865 | 20256887 | 23352000 | -3095113 |
| 3108552 | Brasilândia de Minas | MG | 2509.694 | 4153.8 | 5298 | 22006832.4 | 25430400 | -3423567.6 |
| 3522406 | Itapeva | SP | 1826.258 | 4030.2 | 63073 | 254196804.6 | 302750400 | -48553595 |
| 3521705 | Itaberaí | SP | 1100.247 | 3994.2 | 54504 | 217699876.8 | 261619200 | -43919323 |
| 4114401 | Mangueirinha | PR | 1055.458 | 3974 | 47196 | 187556904 | 226540800 | -38983896 |
|  |  |  |  |  |  |  |  |  |
| *cities with lower average yield* | | | | | | | | |
| 5100359 | Alto Boa Vista | MT | 2248.414 | 3096 | 28035 | 86796360 | 134568000 | -47771640 |
| 5100508 | Alto Paraguai | MT | 1847.354 | 3096 | 3856 | 11938176 | 18508800 | -6570624 |
| 5108303 | União do Sul | MT | 4590.628 | 3096 | 62594 | 193791024 | 300451200 | -106660176 |
| 5105580 | Marcelândia | MT | 12286.12 | 3089.8 | 71171 | 219904155.8 | 341620800 | -121716644 |
| 5107883 | Serra Nova Dourada | MT | 1490.793 | 3084 | 4350 | 13415400 | 20880000 | -7464600 |
| 4101903 | Assaí | PR | 440.347 | 3083.6 | 25499 | 78628716.4 | 122395200 | -43766484 |
| 5000708 | Anastácio | MS | 2913.177 | 3080 | 7534 | 23204720 | 36163200 | -12958480 |
| 4320321 | Senador Salgado Filho | RS | 147.068 | 3076.8 | 10349 | 31841803.2 | 49675200 | -17833397 |
| * Soybean yield average (kg/ha) from 2015/16 to 2020/21 | | | | | |  |  |  |

**Table S4:** Principal component analysis (PCA) for soil attributes.

|  | PC1 | PC2 | PC3 | PC4 | PC5 | PC6 |
| --- | --- | --- | --- | --- | --- | --- |
| Sand | -0.217 | 0.344 | -0.084 | -0.159 | 0.169 | -0.029 |
| Silt | 0.212 | -0.142 | -0.095 | 0.241 | -0.232 | 0.049 |
| Clay | 0.180 | -0.368 | 0.146 | 0.095 | -0.112 | 0.015 |
| Bulk Density | -0.215 | 0.361 | -0.146 | 0.036 | -0.029 | -0.019 |
| SOC | 0.221 | -0.209 | 0.092 | -0.346 | 0.365 | 0.020 |
| SOM | 0.221 | -0.208 | 0.093 | -0.345 | 0.365 | 0.021 |
| pH H_2_O | 0.204 | 0.229 | 0.208 | 0.055 | 0.022 | 0.163 |
| Ca^+2^ | 0.322 | 0.109 | -0.021 | 0.061 | 0.001 | 0.004 |
| Mg^+2^ | 0.302 | 0.108 | -0.083 | 0.122 | -0.014 | -0.046 |
| K^+^ | 0.176 | 0.059 | -0.048 | 0.098 | 0.204 | -0.267 |
| Al ^+3^ | -0.041 | -0.166 | -0.456 | 0.000 | -0.129 | -0.048 |
| H^+^+Al^+3^ | 0.077 | -0.267 | -0.373 | -0.139 | -0.072 | -0.020 |
| CEC pH7 | 0.285 | -0.096 | -0.278 | -0.030 | -0.039 | -0.036 |
| SB | 0.331 | 0.114 | -0.044 | 0.088 | 0.012 | -0.033 |
| V% | 0.249 | 0.281 | 0.134 | 0.101 | -0.003 | -0.023 |
| m% | -0.219 | -0.191 | -0.261 | 0.023 | 0.003 | -0.030 |
| Delta pH | 0.011 | 0.075 | -0.127 | 0.492 | 0.589 | 0.163 |
| Ki | 0.042 | 0.101 | -0.038 | 0.156 | -0.131 | -0.716 |
| Clay activ. | 0.072 | 0.245 | -0.354 | -0.343 | 0.022 | 0.121 |
| Slope | 0.053 | 0.016 | -0.106 | 0.234 | -0.238 | 0.568 |

**Table S5:** Importance of the Principal Component Analysis.

|  |  | PC1 | PC2 | PC3 | PC4 | PC5 | PC6 |
| --- | --- | --- | --- | --- | --- | --- | --- |
| Standard deviation | | 2.81 | 2.00 | 1.78 | 1.18 | 1.12 | 1.04 |
| Proportion of variance | | 0.34 | 0.17 | 0.14 | 0.06 | 0.05 | 0.05 |
| Cumulative proportion | | 0.34 | 0.52 | 0.65 | 0.71 | 0.77 | 0.81 |

***** The proportion of variance (PCn) of each selected principal component is one of the variables for calculation through the SoilPP equation (5).


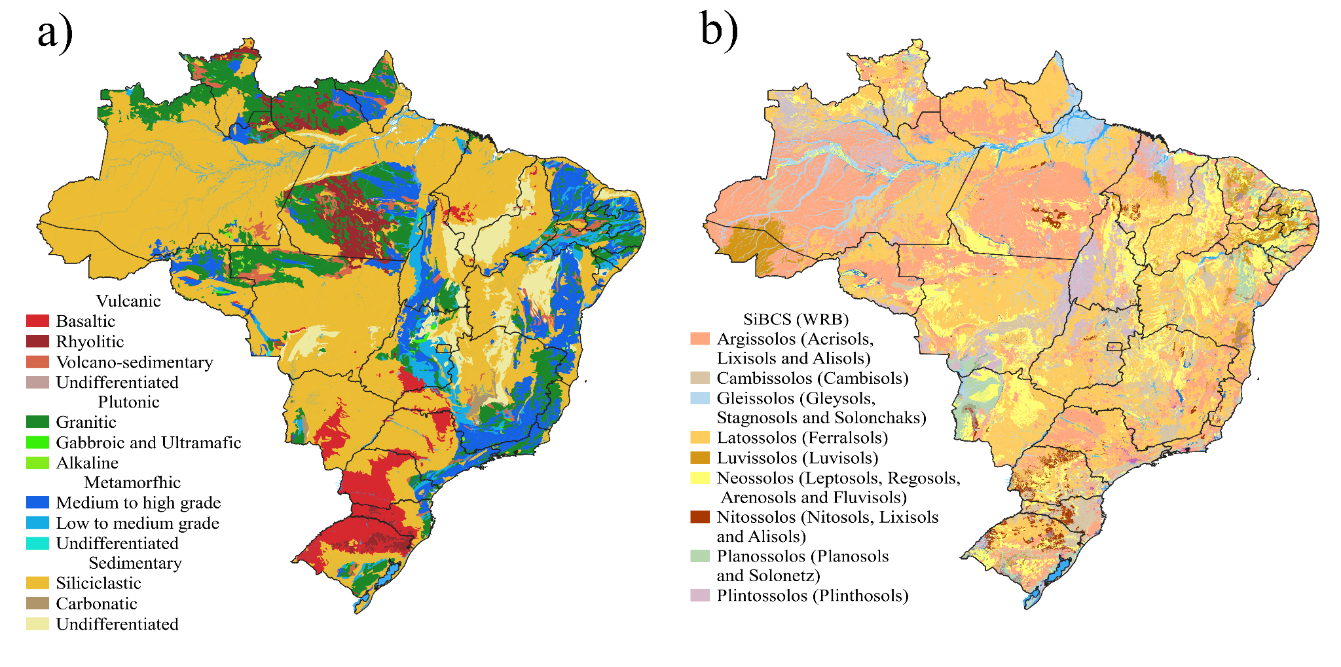
**Fig. S1**. a) Brazilian geological map “scale 1:5.000.000” ^32^. b) Brazilian pedological classification (scale 1:5.000.000) ^19^. The figure was created by the software QGIS Development Team (2021). Version 3.22. http://qgis.osgeo.org and Inkscape Project. (2021). Version 1.1. https://inkscape.org


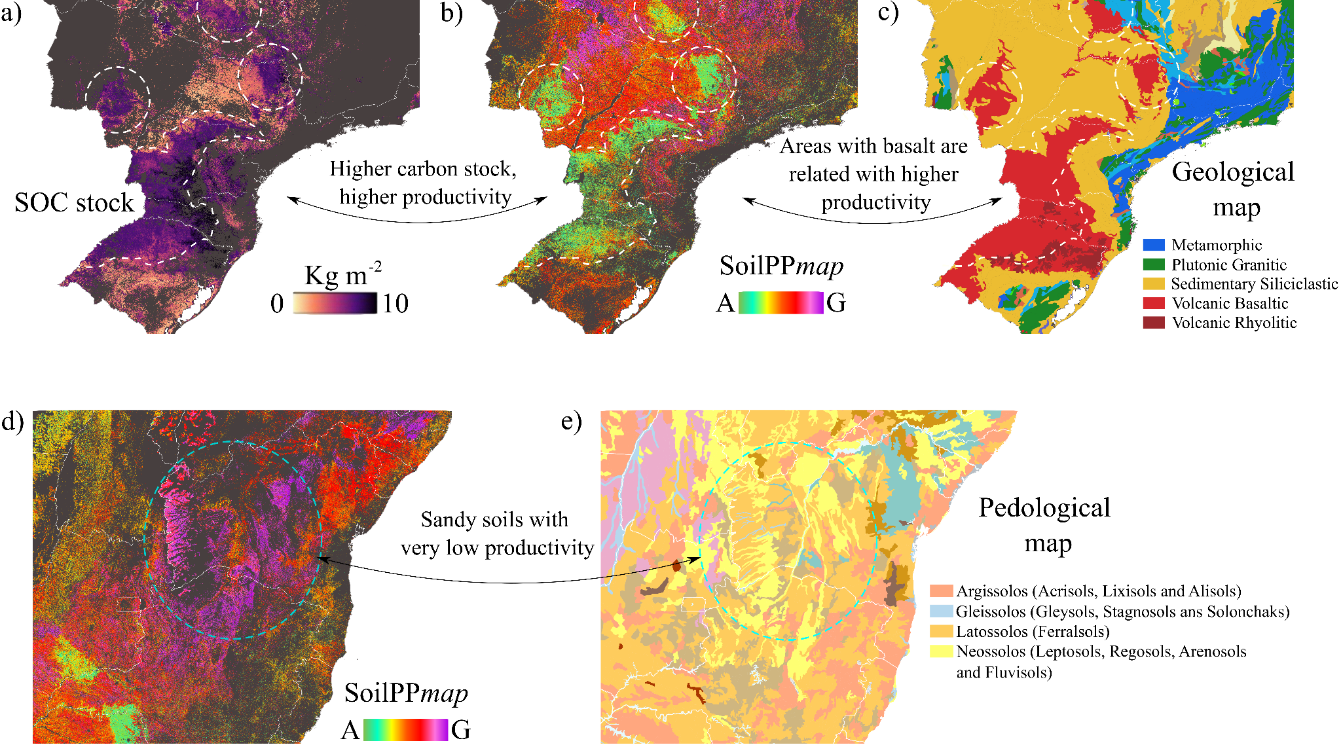


**Fig. S2.** a) SOC stock brazilian map ^37^; b) SoilPP map; c) Brazilian geological map ^32^; d) SoilPP map; e) Brazilian pedological classification ^19^. The figure was created by the software QGIS Development Team (2021). Version 3.22. http://qgis.osgeo.org and Inkscape Project. (2021). Version 1.1. https://inkscape.org

**
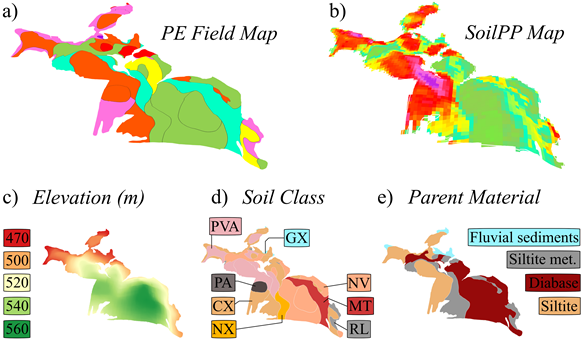
**

**Fig. S3.** a) PES*map* based on pedological classification and sugarcane productivity; b) SoilPP carried out from the evaluation of soil properties (equation 1, 2, 3, 4 and 5) (Table S1 and S2); c) Elevation of the terrain, (meters); d) Pedological classification map represented by soil classes: PVA: Argissolo vermelho amarelo (Acrisols, Lixisols and Alisols); PA: Argissolo amarelo (Acrisols, Lixisols and Alisols); CX: Cambissolo háplico (Cambisols); NX: Nitossolo háplico (Nitisols, Lixisols or Alisols); NV: Nitossolo vermelho (Nitisols, Lixisols or Alisols); MT: Chernossolo argilúvico (Phaeozems, Kastanozems, Chernozems); RL: Neossolo Litólico (Leptosols); e) Parent material. The figure was created by the software QGIS Development Team (2021). Version 3.22. http://qgis.osgeo.org and Inkscape Project. (2021). Version 1.1. https://inkscape.org

**Prediction of SoilPP with Digital Soil Mapping**

The optimal model for each depth (A:0-20; B:40-60 and C:80-100cm) was determined by the Root Mean Square Error (RMSE) of the calibration set after testing all combinations of hyperparameters. The calibration set consisted of bootstrap observations, while the remaining observations that were not sampled were used for testing, resulting in an out-of-bootstrapped validation. The error of the predictive model was assessed using the RMSE, while the explained variance was evaluated by coefficient of determination (R^2^) and the consistency between the predicted values ​​with the variability of the soil dataset by the Performance Ratio to Interquartile Range (RPIQ) was calculated to assess. Final assessment metrics were calculated by averaging the statistics from the test samples, reporting the mean and standard deviation. After the SoilPP maps for each soil layer (A:0-20; B:40-60 and C:80-100cm) were obtained, an average SoilPP map was calculated to represent the SoilPP up to one-meter depth.

The performance of the SoilPP map prediction model for layers A (0-20 cm), B (40-60 cm), and C (80-100 cm) using the covariates extracted through soil samples with geographic coordinates was observed (Table S3). Subsequently this was employed in an algorithm of machine learning (Random forest). The prediction performance for the three layers was with R^2^ less than 0.25, the RMSE ranging from 5.53 to 5.80 points, and the RPIQ greater than 1.41. Layer 'A' presented the lowest R^2^ (0.20), demonstrating the largest size of the forest (FS: 100), where the prediction was calculated using the most unstable hyperparameters of the model, such as the high number of observations on the end sheets (MSL: 500). Layers B and C presented the R^2^ of 0.23 and 0.25. Thus, the R^2^ is higher for subsurface layers. The predictions were calculated from 200 samples in the terminal leaves for both layers B and C. The difference between these layers was in the size of the forest, where layer C had an FS equal to 60, and layer B had an FS equal to 30. The number of hyperparameters of random features tested in each split of the tree was the same for all layers (NRF: 13).

**Table S6.** Prediction performance metrics and optimal hyperparameters of ensembled bootstrapped regression trees used to map the potential productivity of the agricultural soils in Brazil.

| Soil Layers^1^ | FS^2^ | MSL^3^ | NRF^4^ | ^5^ R^2^ | RMSE^6^ | RPIQ^7^ |
| --- | --- | --- | --- | --- | --- | --- |
| A | 100 | 500 | 13 | 0.20 | 5.53 | 1.41 |
| B | 30 | 200 | 13 | 0.23 | 5.80 | 1.90 |
| C | 60 | 200 | 13 | 0.25 | 5.57 | 1.93 |

^1^A: soil layer 0 - 20 cm, B: 40 - 60 cm, C: 80 - 100 cm; ^2^FS: forest size, i.e., number of bootstrapped trees; ^3^NRF: hyper- parameter number of random features tested in each tree split; ^4^MSL: hyper-parameter minimum samples at leaves; ^5^R^2^: coefficient of determination; ^6^RMSE: root mean squared error; ^7^RPIQ: ratio of performance to interquartile range.

The low prediction accuracy of SoilPP map (Table S6) is possibly associated with the evaluation of soil chemical attributes in SoilPP index (Table 1). This issue is linked to the variability of these attributes caused by crop management, such as liming and fertilization of agricultural lands. This was observed by Safanelli et al. (2021) performing poor prediction of soil pH_H2O_ (R^2^ 0.07) and CEC mmolc kg ^-1^ (R^2^ 0.27) for Brazilian agricultural areas using approximately 4500 soil samples. Also, Mendes et al. (2019) performed the prediction of chemical attributes such as CEC (R^2^ 0.35 to 0.02), base saturation (R^2^ 0.12 to 0.05), aluminum saturation (R^2^ 0.27 to 0.17), and Al (R^2^ 0.36 to 0.07). The authors used different covariates and prediction methods, but the model accuracy was also low. Poor chemical property prediction performance also occurred in other studies (Poppiel et al., 2019; Rizzo et al., 2020). Therefore, the insertion of chemical attributes (Table 1) in the SoilPP index to generate the SoilPP map was probably the main factor that reduced the accuracy of the prediction, since it is one of the pillars that influence on the index result (Table S4) and present high variability in the landscape.
